# Drug‐drug interactions between feminizing hormone therapy and pre‐exposure prophylaxis among transgender women: the iFACT study

**DOI:** 10.1002/jia2.25338

**Published:** 2019-07-12

**Authors:** Akarin Hiransuthikul, Rena Janamnuaysook, Kanittha Himmad, Stephen J Kerr, Narukjaporn Thammajaruk, Tippawan Pankam, Kannapat Phanjaroen, Stephen Mills, Ravipa Vannakit, Praphan Phanuphak, Nittaya Phanuphak, the iFACT Study Team

**Affiliations:** ^1^ PREVENTION Thai Red Cross AIDS Research Centre Bangkok Thailand; ^2^ HIV‐NAT Thai Red Cross AIDS Research Centre Bangkok Thailand; ^3^ Faculty of Medicine Chulalongkorn University Bangkok Thailand; ^4^ LINKAGES Thailand FHI 360 Bangkok Thailand; ^5^ Office of Public Health United States Agency for International Development Bangkok Thailand

**Keywords:** drug‐drug interactions, feminizing hormone, pre‐exposure prophylaxis, prevention, transgender women

## Abstract

**Introduction:**

Concerns over potential drug‐drug interactions (DDI) between feminizing hormone therapy (FHT) and pre‐exposure prophylaxis (PrEP) have hampered uptake and adherence of PrEP among transgender women (TGW). To determine DDI between FHT and PrEP, we measured the pharmacokinetic (PK) parameters of blood plasma estradiol (E2) and tenofovir (TFV) in Thai TGW.

**Methods:**

Twenty TGW who never underwent orchiectomy and had not received injectable FHT within six months were enrolled between January and March 2018. FHT (E2 valerate and cyproterone acetate) were prescribed to participants at baseline until week 5, and then from week 8 until the end of study. Daily PrEP (tenofovir disoproxil fumarate/emtricitabine) was initiated at week 3 and continued without interruption. Intensive E2 PK parameters and testosterone concentration at 24 hours (C_24_) were measured at weeks 3 (without PrEP) and 5 (with PrEP), and intensive TFV PK parameters were measured at weeks 5 (with FHT) and 8 (without FHT).

**Results:**

Median (interquartile range) age, body mass index, and creatinine clearance were 21.5 (21–26) years, 20.6 (19.0‐22.4) kg/m^2^, and 116 (101‐126.5) mL/min, respectively. The geometric mean (%CV) of area under curve from time zero to 24 hours (AUC_0‐24_), maximum concentration (C_max_), and C_24_ of E2 at weeks 3 and 5 were 775.13 (26.2) pg h/mL, 51.47 (26.9) pg/mL, and 15.15 (42.0) pg/mL; and 782.84 (39.6), 55.76 (32.9), and 14.32 (67.4), respectively. The geometric mean (%CV) of TFV AUC_0‐24_, C_max_, and C_24_ at weeks 5 and 8 were 2242.1 (26.5) ng h/mL, 353.9 (34.0) ng/mL, and 40.9 (31.4) ng/mL; and 2530.2 (31.3), 311.4 (30.0), and 49.8 (29.6), respectively. The geometric mean of TFV AUC_0‐24_ and C_24_ at week 5 were significantly less than that at week 8 by 12% (*p* = 0.03) and 18% (*p* < 0.001), respectively. There were no significant changes in E2 PK parameters and median C_24_ of bioavailable testosterone between week 3 and week 5.

**Conclusions:**

Our study demonstrated lower blood plasma TFV exposure in the presence of FHT, suggesting that FHT may potentially affect PrEP efficacy among TGW; but E2 exposure was not affected by PrEP. Further studies are warranted to determine whether these reductions in TFV are clinically significant.

Clinical Trial Number: NCT03620734.

## 1 Introduction

Transgender women (TGW) are heavily burdened by HIV infection, with an estimated prevalence of 19.1% and a 49‐fold increased risk of HIV acquisition compared to general population 1. While studies often subsume TGW within the men who have sex with men (MSM) population, TGW typically do not consider themselves as MSM, and hold unique and distinct medication exposure, social and behavioural characteristics compared to MSM, including lower education, lower income levels, higher proportion of engaging in sex work, and the use of feminizing hormone therapy (FHT) 2, 3, 4.

Considered as the standard of care for TGW, the goal of FHT is to induce secondary female sex characteristics while reducing male sex characteristics 5, 6. This usually involves the use of an estrogen and anti‐androgen concomitantly. In a cross‐sectional sample of TGW in Thailand, approximately 75% reported using FHT for a mean duration of 10 years 7. Many TGW used higher‐than‐recommended doses and 50% reported symptoms from these higher doses of oral contraceptive pills 8. In another study among TGW sex workers in Bangkok, 80% reported current hormone use, and 50% used injectable hormones 9.

The use of emtricitabine (FTC) and tenofovir disoproxil fumarate (TDF) for pre‐exposure prophylaxis (PrEP) is effective in reducing HIV transmission among MSM, heterosexual men and women, and people who inject drugs 10, 11, 12. Unfortunately, only a small number of TGW have been included in clinical trials of PrEP, and the results have been disappointing. The iPrEx trial reported that PrEP showed no efficacy in preventing HIV infection among TGW in an intention‐to‐treat analysis population, TGW were more likely to have undetectable drug levels compared to non‐TGW participants, and none of the TGW who seroconverted had detectable levels of tenofovir (TFV) 13. Two potential explanations for this are that (1): TGW in PrEP trials had good adherence to PrEP, but drug‐drug interactions (DDI) between feminizing hormones and PrEP resulted in lower concentrations of PrEP among TGW; or, (2): TGW actually had poorer adherence to PrEP than non‐TGW participants, potentially because they prioritized the use of FHT over HIV medications due to concerns over potential DDIs. Such concerns on the part of TGW have been noted in numerous surveys 14, 15, 16, 17. In either case, the potential for DDI appears to contribute to low efficacy of PrEP among TGW, and the actual presence of DDI between FHT and PrEP medications remains an important issue to be explored.

Many antiretroviral (ARVs) agents, specifically non‐nucleoside reverse transcriptase inhibitors and protease inhibitors, are metabolized through the cytochrome P450 (CYP) system allowing for interference with the metabolism of several drugs eliminated by CYP isoenzymes, including estrogens and cyproterone acetate (CPA). Because most studies on interactions between ARVs and estrogens are in the context of contraception, ethinyl estradiol (E2), rather than 17β E2 (the recommended form of estrogen in FHT), is the drug used in these studies. Based on available data, there are no known interactions between nucleos(t)ide reverse transcriptase inhibitors and estrogens. Currently, there are no published literature on DDI between FHT and PrEP among TGW. It is also important to note that, in addition to recommending a different form of estrogen than what is prescribed for contraception, FHT guidelines typically recommend a much higher dose of estrogen than is recommended for treatment of menopause 18. We report here on the findings of the iFACT (Interaction between the use of FHT and Antiretroviral agents Concomitantly among TGW) study. The objective of this study is to investigate potential DDI between the hormones used in FHT and the ARV agents used for PrEP among TGW in Thailand.

## 2 Methods

### 2.1 Enrolment and study population

The Thai Red Cross AIDS Research Centre's (TRC‐ARC) Tangerine Community Health Clinic is among the first transgender‐specific sexual health clinics in Asia. Managed by trained transgender staff and gender‐sensitive medical professionals, Tangerine Community Health Clinic has already served more than 2500 TGW since its opening in November 2015. According to our data, TGW usually take hormones based on their transgender peers’ experience and, if affordable, many also undergo breast augmentation and facial feminization surgery. Decisions on genital surgery depends on many factors, including cost and occupation, since pre‐operative TGW are more popular in sex work industry in Thailand. Among 2500 TGW clients, 20% have undergone genital surgery. Sex reassignment surgery (SRS) is neither include in the universal health coverage nor the private health insurance in Asia region.

The iFACT prospectively screened and enrolled 40 TGW at the Tangerine Community Health Center, which is managed by the TRC‐ARC in Bangkok, Thailand. Twenty HIV‐negative TGW were enrolled to determine DDI between FHT and PrEP and 20 HIV‐positive TGW are currently being enrolled to determine DDI between FHT and Antiretroviral therapy (ART). This article presents and discusses data from the 20 HIV‐negative TGW.

HIV‐negative TGW were enrolled between January and March 2018. Eligibility criteria included being of Thai nationality, aged 18‐40 years, with body mass index (BMI) of 18.5‐24.9 kg/m^2^, were ARV‐naïve had calculated creatinine clearance (CrCl) ≥ 60 mL/min using Cockcroft‐Gault equation, and had alanine aminotransferase (ALT) ≤ 2.5 × upper limit of normal. Despite that there are no previous studies among TGW, associations between sex hormones and BMI in non‐TGW population have been reported 19, 20. To eliminate potential confounders in the setting of this intensive pharmacokinetic (PK) study, BMI was included in the inclusion criteria recruiting only those in the normal range (18.5‐24.9 kg/m^2^), according to the World Health Organization. Exclusion criteria were TGW with a history of allergy to the hormonal compounds used in this study who had undergone orchiectomy, received injectable FHT in the previous six months, or were currently using a medication with possible DDI with FHT and/or PrEP. Injectable FHT can have a half‐life of up to 10 days which would interfere our PK assessment at week 3. In contrast, other forms of FHT such as transdermal, oral, and sublingual, have a half‐life of approximately one‐two days, so the elimination of these forms of FHT would occur before our PK assessment at week 3, and therefore, were permitted in our study. The study (NCT03620734) was approved by the institutional review board of the Faculty of Medicine, Chulalongkorn University, Bangkok, Thailand (IRB No. 525/60b) and all participants gave informed consent.

### 2.2 Recommendations for FHT

The Tangerine Community Health Center recommends FHT according to its Gender Affirmative Hormone Therapy Protocol. The protocol was developed based on the World Professional Association for Transgender Health Standard of Care for the Health of Transexual, Transgender and Gender Nonconforming People version 7, the Center of Excellence for Transgender Health Guidelines for the Primary and Gender‐Affirming Care for Transgender and Gender Nonbinary People, and the Asia Pacific Transgender Network Blueprint for the Provision of Comprehensive Care for Trans People and Trans Communities in Asia and the Pacific, with particular modifications made specifically for Asian transgender individuals 21, 22, 23. Our recommendations suggest the use of status of SRS and body hair patterns as key considerations when prescribing FHT regimens. The options for estrogen according to our guidelines were E2 valerate, oral E2 hemihydrate, and E2 hemihydrate patch; and the options for anti‐androgen were CPA and spironolactone.

### 2.3 Study drugs

The selection of FHT used in this study was based on the Tangerine Community Health Centre's protocol as well as the availabilities and popularities of FHT regimens in our TGW community. E2 valerate and CPA are the most commonly used estrogen and anti‐androgen in the FHT regimen, respectively, among Thai TGW since they are available as over‐the‐counter medications and do not require a physician's prescription. Therefore, all participants were placed on the same regimen consisting of E2 valerate 2 mg (Progynova^®^; Delpharm Lille S.A.S., Lyz Lez Lannoy, France) and CPA 25 mg (Androcur^®^; Bayer Weimar GmbH und Co. KG, Weimar, Germany) once daily for FHT in our study. Generic TDF/emtricitabine (Teno‐Em^®^; Thai GPO, Bangkok, Thailand) were used for daily PrEP.

### 2.4 Study procedure

Our study aimed to do a two‐way analysis of DDI to show the effect of PrEP towards FHT and vice versa. Therefore, our study was designed to measure the intensive PK parameters during the period in which the participants to receive FHT only, then FHT plus PrEP, then PrEP only in a chronological order.

**Figure 1:**
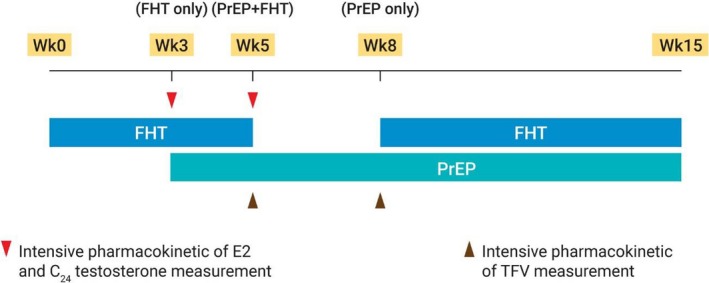
iFACT study scheme FHT was initiated at week 0 until week 5, and later restarted at week 8 until the completion of study period. PrEP containing TFV disoproxil fumarate and emtricitabine was initiated at week 3 and continued without interruption until the completion of study period. Intensive PK parameters of E2 were assessed at week 3 (FHT only) and week 5 (PrEP and FHT). Intensive PK parameters of TFV were assessed at week 5 (PrEP and FHT) and week 8 (PrEP only). Testosterone concentration at 24 hours (C_24_) was also measured at week 3 and week 5 to indirectly determine the anti‐androgen effect of FHT. C_24_, concentration at 24 hours; E2, estradiol; FHT, feminizing hormone therapy; iFACT, Interaction between the use of FHT and Antiretroviral agents Concomitantly among TGW; PK, pharmacokinetics; PrEP, pre‐exposure prophylaxis; TFV, tenofovir.

FHT was prescribed to participants at baseline. The first intensive PK parameters of E2 were measured at week 3 to assess E2 levels without the presence of PrEP. After blood tests for E2 PK parameters were done, PrEP was initiated at the same visit. The second intensive PK parameters of E2 and the first intensive PK parameters of TFV were measured at week 5 to assess E2 and TFV levels in the presence of both FHT and PrEP. After blood tests for E2 and TFV PK parameters were done, FHT was discontinued at the same visit. The second intensive PK parameters of TFV were then measured at week 8 to assess TFV levels without the presence of FHT. After blood tests for TFV PK parameters were done, FHT was then restarted and continued until the completion of the 15‐week study period for safety evaluation. Besides intensive PK parameters of E2 and TFV, testosterone concentration at 24 hours (C_24_) was measured at week 3 and week 5 to indirectly determine the anti‐androgen effect of CPA. All participants received daily reminder by our study staff to ensure their adherences to PrEP and FHT as prescribed. The iFACT study scheme is demonstrated in Figure 1.

To ensure adherence to study drugs and prevent the use of other feminizing hormones products, all participants received daily reminders from our staff member between each visit via a social communication application of their choice, to adhere to study drugs and not use any other feminizing hormones. In addition, pill counts and self‐reported use of other medications were assessed at each visit.

### 2.5 PK methods

A 24‐hour PK study was performed for E2 and TFV. On PK day, plasma was collected at 0 (pre‐dose), 1, 2, 4, 6, 8, 10, 12 and 24 hours after directly observed medication ingestion with a standardized meal of 1.8 kcal (a total of 9 samples). No other food was allowed.

#### 2.5.1 PK analysis of TFV

Lithium heparin blood samples were mixed and sent to the HIV‐NAT research collaboration laboratory in ambient condition immediately for PK analysis of TFV. Blood samples were centrifuged at 1800 g (3000 rpm) for 10 minutes at 20°C. Plasma was separated and transferred to labelled polypropylene tubes and stored at −20°C until analysis. Total collection sample process was performed within two hours after collection. TFV Plasma concentrations were determined by a validated high‐performance liquid chromatography with fluorescence detection method 24, with a lower limit of quantification of 15 ng/mL. The TFV calibration curve was linear over the concentration range of 15 to 1500 ng/mL. The within‐run and between‐run %variation (precision) was <10% and the %accuracy of the ARVs was between 95% and 105%.

#### 2.5.2 PK analysis of E2

Clotted blood was collected using clot blood tube and sent immediately to the HIV‐NAT laboratory for serum separation. Blood samples were centrifuged at 1800 g (3000 rpm) for 10 minutes at 20°C. Serum was separated and transferred to labelled polypropylene tubes and stored at −20°C until analysis (less than six months). E2 was performed by Elecsys Estradiol III Assay (Roche Diagnostics GmbH, Mannheim, Germany) based on the electrocheminescence immunoassay technique. The limits of lower and upper detection were 5 and 3000 pg/mL, respectively (18.4‐11,010 pmol/L).

In addition to intensive PK analysis of TFV and E2, C_24_ of testosterone was measured at week 3 and week 5 to indirectly assess DDI between PrEP and FHT by comparing the anti‐androgen effect of CPA before and after the presence of PrEP.

### 2.6 Statistical analysis

Statistical and PK analysis was performed using Stata 15.1 (StataCorp LP, College Station, TX, USA). Demographic and laboratory assessment were described by frequencies, percentages, median, and interquartile range (IQR), as appropriate. For the PK assessments of E2 and TFV, the following parameters were derived using non‐compartmental models: area under curve from time zero to 24 hours (AUC_0‐24_), maximum concentration (C_max_), and C_24_. The geometric mean and % coefficient of variation were summarized at each study week. Generalized estimating equations were used to assess the change in the geometric mean of AUC_0‐24_, C_max_ and C_24_ to calculate the geometric mean ratios (GMRs) for E2 and TVF; the reference week was the point at which the E2 or TFV was given alone. Changes in bioavailable testosterone, ALT and other laboratory parameters from baseline to subsequent study weeks were tested with a Wilcoxon sign rank test. Median concentrations at each time point were used to plot a concentration time curve, by study week. Associations between CrCl and E2 or TFV AUC_0‐24_ and C_24_ were assessed by Spearman's rank correlation coefficient.

## 3 Results

A total of 20 HIV‐negative TGW were enrolled in this analysis. The median (IQR) age, BMI, CrCl and ALT levels were 21.5 (21‐26) years, 20.6 (19.0‐22.4) kg/m^2^, 116 (101‐126.5) mL/min, and 13.5 (9.0‐19.5) IU/l, respectively. All participants reported 100% adherence to PrEP and FHT as prescribed.

### 3.1 PK parameters

#### 3.1.1 Feminizing hormones levels

**Figure 2:**
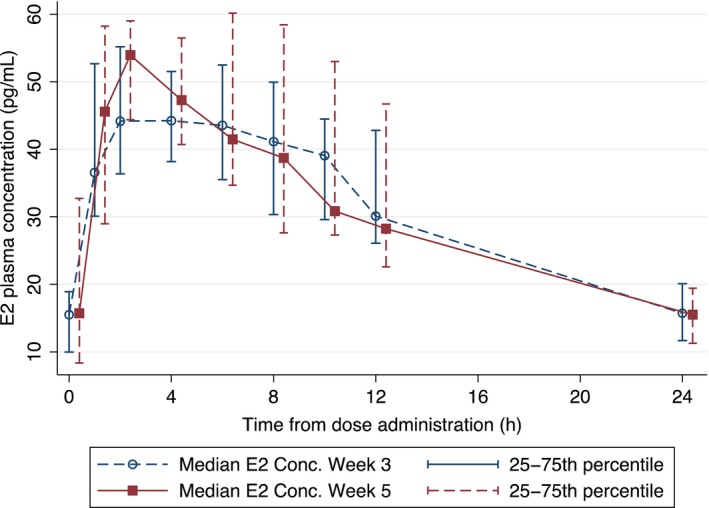
Median estradiol (E2) concentration‐times curves at week 3 and week 5 Error bars represent the 25th to 75th percentile. Week 5 times have been offset by 0.4 hours to improve readability.

The geometric mean (%CV) of AUC_0‐24_, C_max_, and C_24_ of E2 at weeks 3 (FHT only) and 5 (PrEP and FHT) were 775.13 (26.2) pg h/mL, 51.47 (26.9) pg/mL, and 15.15 (42.0) pg/mL; and 782.84 (39.6), 55.76 (32.9), and 14.32 (67.4), respectively (Table 1). The median blood plasma concentration‐time profiles of E2 are plotted in Figure 2.

**Table 1:** Summary of geometric mean (%CV) of E2 and TFV PK parameters

E2 PK parameter	Week 3 (FHT)	Week 5 (PrEP + FHT)	GMR (95% CI)	*p*‐Value
AUC_0‐24_ (pg h/mL)	775.13 (26.2)	782.84 (39.6)	1.01 (0.89‐1.15)	0.88
C_max_ (pg/mL)	51.47 (26.9)	55.76 (32.9)	1.08 (0.94‐1.24)	0.25
C_24_ (pg/mL)	15.15 (42.0)	14.32 (67.4)	0.95 (0.75‐1.19)	0.63

TFV PK parameter	Week 5 (PrEP + FHT)	Week 8 (PrEP only)	GMR (95% CI)	*p*‐Value
AUC_0‐24_ (ng h/mL)	2242.1 (26.5)	2530.2 (31.3)	0.88 (0.79‐0.99)	0.03
C_max_ (ng/mL)	353.9 (34.0)	311.4 (30.0)	1.14 (0.97‐1.33)	0.10
C_24_ (ng/mL)	40.9 (31.4)	59.8 (29.6)	0.82 (0.76‐0.89)	<0.001

There were no significant changes in E2 PK parameters between week 3 and 5. The geometric mean of AUC_0‐24_ and C_24_ of TFV at week 5 were significantly less than that at week 8 by 12% (*p* = 0.03) and 18% (*p* < 0.001), respectively.

AUC_0‐24_, area under curve from time zero to 24 hours; C_max_, maximum concentration; C_24_, concentration at 24 hours; E2, estradiol; FHT, feminizing hormone therapy; GMR, geometric mean ratio; PK, pharmacokinetics; PrEP, pre‐exposure prophylaxis; TFV, tenofovir.

There were no significant changes in E2 PK parameters between weeks 3 and 5. Median (IQR) C_24_ of bioavailable testosterone was comparable between weeks 3 and 5 (0.10 (0.04‐0.18) vs. 0.08 (0.03‐0.15) ng/mL, *p* = 0.71).

#### 3.1.2 TFV levels

**Figure 3:**
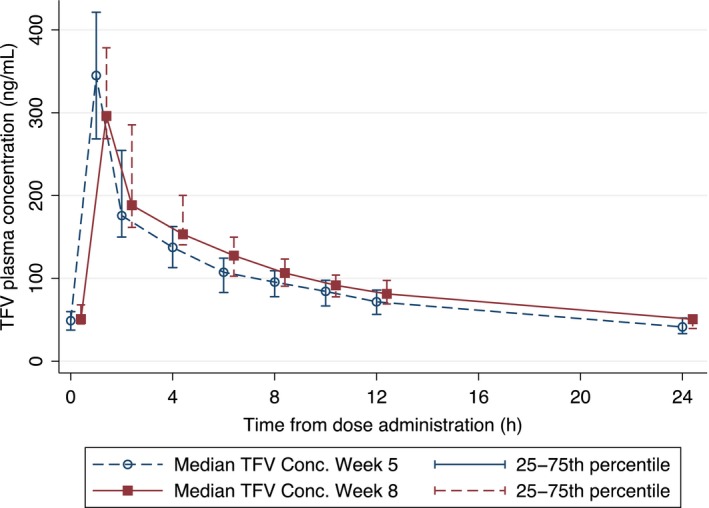
Median tenofovir (TFV) concentration‐times curves at week 5 and week 8 Error bars represent the 25th to 75th percentile. Week 8 times have been offset by 0.4 hours to improve readability.

The geometric mean (%CV) of TFV AUC_0‐24_, C_max_, and C_24_ at weeks 5 (PrEP and FHT) and 8 (PrEP only) were 2242.1 (26.5) ng h/mL, 353.9 (34.0) ng/mL, and 40.9 (31.4) ng/mL; and 2530.2 (31.3), 311.4 (30.0), and 49.8 (29.6), respectively (Table 1). The geometric mean of TFV AUC_0‐24_ and C_24_ at week 5 were significantly less than that at week 8 by 12% (*p* = 0.03) and 18% (*p* < 0.001), respectively. The median (IQR) time to C_max_ of TFV was one hour at both study visits. The mean plasma concentration‐time profiles of TFV are plotted in Figure 3.

Of 20 TGW, 14 (70%) TGW had any reductions in C_24_ of TFV in the presence of FHT, with the median (IQR) reduction of 9 (7‐13) ng/mL. All TGW had detectable C_24_ of TFV in both week 5 and 8. GMRs adjusted for weight at the time of each assessment were examined in a sensitivity analysis, and showed the GMR C_24_ and C_max_, their precision reflected by the 95%CI, and their significance level were identical after adjustment for weight. The GMR for TFV AUC_0‐24_ was higher by 1 percent after adjustment for weight (0.89 (0.80‐0.99), *p* = 0.04).

### 3.2 Safety

There were no study‐related adverse events during the study period. The median (IQR) CrCl at weeks 3, 5 and 8 was 145.7 (121.1 to 155.2), 137.9 (119.6 to 159.8) and 131.6 (116.0 to 143.1) mL/min, respectively. We found no significant correlation between CrCl and E2 AUC_0‐24_ or C_24_ at weeks 3 or 5, or between CrCl and TFV AUC_0‐24_ and C_24_ at weeks 5 or 8 (Table S1). At 15 weeks, there was a significant change from entry to week 15 in median (IQR) ALT (13.5 (9.0‐19.5) to 19.5 (14‐22) IU/l, *p* = 0.02), but no significant change in CrCl was observed (116 (101‐126.5) to 117 (90.1‐103.8) mL/min, *p* = 0.76). All participants remained HIV‐negative.

## 4 Discussion

Our study is among the first to determine DDI between the use of FHT and PrEP among TGW. In this two‐way analysis, we found that blood plasma E2 exposure was not significantly affected by the presence of PrEP; however, blood plasma TFV exposure was significantly reduced by 12% in the presence of FHT.

Concerns over potential DDI between FHT and PrEP have hampered uptake and adherence of PrEP among TGW, since TGW often prioritize FHT over PrEP 16, 17. This is unfortunate as studies have shown that many TGW have positive attitudes towards PrEP and are interested in using PrEP 25, 26, 27. The lack of information on this topic makes counselling for HIV prevention among TGW challenging.

Although concluding a mechanism for the interaction is difficult, we hypothesize that DDI between FHT and PrEP involves altered drug clearance. As shown in Figure 3, the C_max_ is extremely variable, but the median TFV levels are higher at each time point when the drug is given in the absence of E2, suggesting the clearance is altered. Moreover, the median (IQR) time to C_max_ of TFV was one hour at both study weeks, suggesting that the absorption was not altered by the co‐administered E2.

To our knowledge, there is currently no published literature addressing DDI between FHT and PrEP among TGW. Among the most recent data – presented by Cottrell et al. at the 22nd International AIDS Conference and Hendrix et al. at the HIVR4P in 2018 – found an altered TDF/FTC pharmacology in a TGW cohort. For the former study, FHT – consisting of E2, medroxyprogesterone, and spironolactone – was given to four HIV‐positive TGW and four cis‐gender women (CGW). The results from rectal tissue biopsy showed a significantly lower level of active metabolite TFV diphosphate to deoxyadenosine triphosphate ratio among TGW compared to CGW (2.5 vs. 19.6, *p* < 0.05), suggesting that FHT may impact PrEP efficacy 28. For the latter study, TDF/FTC levels were compared between eight TGW who were already taking FHT – all were taking E2 or E2 analogues but in different combinations – and cis‐gender men (CGM) after TDF/FTC was given for seven days. The results showed significantly lower trough concentration of plasma TFV (20%, *p* = 0.02) and FTC (26%, *p* = 0.03) and AUC_0‐24_ of plasma FTC (24%, *p* = 0.03) among TGW compared to CGM, also suggesting that FHT may impact PrEP efficacy 29.

Studies on DDI between hormones use and ARV agents usually were performed in the context of contraception among CGW with the ultimate goal of assessing either PK outcomes and/or clinical outcomes. Todd et al. studied DDI between levonorgestrel implants and TDF/FTC among 29 healthy CGW in Kenya and reported no significant differences in levonorgestrel levels between those taking TDF/FTC and placebo 30. Another study from the US also demonstrated no significant changes in norelgestromin and ethinyl E2 levels in CGW taking TDF 31. Furthermore, many studies have shown similar contraceptive outcomes between CGW using hormonal contraception who take PrEP (both TDF and TDF/FTC) or placebo 30, 32, 33. Our findings similarly demonstrated no significant changes in blood hormones exposure in the presence of PrEP, suggesting that TGW can confidently use PrEP without concerns of diminishing feminizing effects due to sub‐optimal E2 levels.

Among the scarce data describing the effects of hormonal contraception towards a TDF/FTC PrEP regimen, Kearney and Mathias demonstrated comparable blood TFV levels among CGW in the presence of norelgestromin and ethinyl E2 when compared to historical data 31. In a large cohort of HIV‐serodiscordant couples consisting of over 2300 HIV‐negative women, HIV protection outcome using TDF/FTC, compared with placebo, did not differ statistically for women using depot medroxyprogesterone acetate and women using no hormonal contraception 34. Our study demonstrated significantly lower AUC_0‐24_ and C_24_ of TFV levels (by 12% and 18%, respectively) in the presence of FHT, confirming that there are potential DDIs between FHT containing E2 valerate and CPA and PrEP among TGW. Although we were unable to conclude to what extent these reductions could impact on PrEP efficacy among TGW, in a previous study detection of plasma TFV concentrations were highly predictive of protection from HIV infection regardless of the specific cut‐off levels 35; all participants in our study had detectable plasma concentration of TFV in both the absence and presence of FHT.

Although we demonstrate a potential DDI between FHT and PrEP, which is one of the major concerns for TGW, other factors are also important in increasing PrEP uptake and retention among TGW. One important component in TGW holistic care is the integration of FHT services into HIV and sexually transmitted infections services. The value of such integration was demonstrated by recent programmatic data collected among a group of more than 900 TGW accessing clinic‐based sexual health services in Thailand. TGW clients who accessed FHT services integrated into a comprehensive sexual health package were more likely than other TGW clients to re‐visit the clinic, repeat syphilis testing, repeat HIV testing, and to access PrEP services 36. Findings generated through the current study can be used to counsel TGW clients on concurrent use of FHT and PrEP that PrEP does not have a negative impact on efficacy of E2 valerate and CPA used orally for FHT. FHT, however, may lower PrEP level in blood although currently there is no data to point to any clinical significances in HIV prevention efficacy. Since the 12% reduction in AUC_0‐24_ and 18% reduction in C_24_ seen in our study are similar to the 14% reduction which would be seen in one missed dose weekly, a further reduction associated with the on‐demand regimen may not provide the same level of protection that it does in cisgender men; therefore, TGW should be supported to achieve the highest level of daily PrEP adherence and not to offer the on‐demand PrEP regimen.

While our study provided novel data on this previously unknown DDI between FHT and PrEP among TGW, certain limitations need to be considered. We did not use a randomized crossover design so a period effect was possible. However, the half‐lives of the drugs studied, and the periods between intensive PK sampling assured both that steady‐state conditions were achieved for each drug of interest, and that elimination of the drug dosed in the prior period was completed. Although we used several measures to ensure adherence to study drug and prevent the use of other feminizing hormones, dosing was not directly observed between visits and non‐use of other feminizing products was only checked by self‐report, not with a biological test, including E2 levels at week 8. We only measured blood TFV levels and not the active intracellular form; thus, the reduction in TFV levels might not reflect the actual reduction in PrEP efficacy, especially at the target tissues where HIV first comes into contact with the host cells in most cases. In addition, we did not measure FTC and CPA levels due to the unavailability of the laboratory test in our facility. Although clinical trials up till now have reported no significant differences between the protective effect of TDF alone and TDF/FTC against HIV acquisition among HIV‐negative heterosexual men and women 37, 38, there are still concerns over the protective effect of TDF alone among MSM 39. Further studies are warranted to measure the FTC levels in TGW. Instead of measuring CPA, we opted to measure testosterone levels as an indirect measurement of CPA activities 40. Moreover, using a specific FHT regimen in this study limits the generalizability of findings to TGW who use different FHT regimens. Lastly, due to the nature of an intensive PK study design, all participants enrolled in our study had very homogenous characteristics. Therefore, we were unable to conclude whether there is any DDI between PrEP and FHT among TGW with ethnicities, races, ages, or BMI that differ from those of our study participants.

## 5 Conclusions

Our study demonstrated no significant changes in blood E2 exposure in the presence of PrEP, suggesting that TGW can use FHT and PrEP concurrently without the concerns that PrEP will diminish the feminizing effects from FHT. However, blood TFV exposure was decreased by 12% in the presence of FHT, suggesting that FHT may potentially affect PrEP efficacy among TGW. Further studies are warranted to determine whether these reductions in TFV also occur in target tissue, different FHT regimens, and are clinically significant regarding the HIV protective effect of PrEP among TGW.

## Competing interests

All authors declare no competing interests related to this work.

## Authors’ contributions

AH drafted the manuscript and study protocol. AH and NP led the study. KH and RJ coordinated the study and oversaw the participants. AH, SK, NT, PP and NP participated in study design. SK performed all statistical analysis. NT and TP performed laboratory testing. NP initiated the concept for the study. SM and RV approved the study initiation. All authors critically reviewed and approved the final draft of manuscript.

## Supporting information


**Table S1.** Spearman's correlation coefficients (ρ, and *p*‐value in parentheses) between CrCl and E2 or TFV AUC_0‐24_ and C_24_ at different study weeks.
