# Supplementary material for: Drug‐drug interactions between feminizing hormone therapy and pre‐exposure prophylaxis among transgender women: the iFACT study
Source: J Int AIDS Soc. 2019 Jul 12;22(7):e25338. doi: 10.1002/jia2.25338 (PMC6625338; doi:10.1002/jia2.25338)
Supplement: Supplementary file 1 — Table S1. Spearman's correlation coefficients (ρ, and p‐value in parentheses) between CrCl and E2 or TFV AUC0‐24 and C24 at different study weeks. [file JIA2-22-e25338-s001.docx]

**Supplementary Table 1.** Spearman’s correlation coefficients (rho, and p-value in parentheses) between CrCl and E2 or TFV AUC_0-24_ and C_24_ at different study weeks.

| Week | E2 AUC_0-24_ | E2 C_24_ | TFV AUC_0-24_ | TFV log_e_ C_24_ |
| --- | --- | --- | --- | --- |
| Week 3 | 0.31 (0.20) | 0.34 (0.15) |  |  |
| Week 5 | -0.21 (0.38) | -0.12 (0.62) | -0.36 (0.13) | -0.35 (0.14) |
| Week 8 |  |  | -0.17 (0.49) | -0.19 (0.43) |

Abbreviations: AUC_0-24_, area under curve from time zero to 24 hr; C_24_, concentration at 24 hr; E2, estradiol; CrCl, creatinine clearance; TFV, tenofovir
